# A novel anthropometric method to accurately evaluate tissue deformation

**DOI:** 10.3389/fbioe.2025.1632806

**Published:** 2025-07-28

**Authors:** Chongyang Ye, Xiaolu Li, Haiyan Song, Yu Shi, Ruixin Liang, Jun Zhang, Ka Po Lee, Zhaolong Chen, Beibei Zhou, Raymond Kai-Yu Tong, Kit-Lun Yick, Sun-Pui Ng, Joanne Yip

**Affiliations:** ^1^School of Fashion and Textiles, The Hong Kong Polytechnic University, Hong Kong, China; ^2^Medical Imaging Department, Shenzhen Second People’s Hospital, Shenzhen, China; ^3^ College of Arts, South China Agricultural University, Guangzhou, Guangdong, China; ^4^ Department of Biomedical Engineering, The Chinese University of Hong Kong, Shatin, Hong Kong SAR, China; ^5^ School of Professional Education and Executive Development, The Hong Kong Polytechnic University, Hung Hom, Hong Kong SAR, China

**Keywords:** anthropometric method, body scanning/imaging, soft tissue deformation, sportswear design, analytical model

## Abstract

**Introduction:**

Biomechanical imaging through body scanning can provide a more comprehensive understanding of the soft tissue deformation exerted by compression sportswear, which is crucial in sports science research and functional sportswear design. However, displacement from movement affects alignment so accurately measuring tissue deformation with different wear conditions becomes challenging.

**Methods:**

To address this issue, an analytical model is constructed to predict tissue deformation by using the Boussinesq solution, which is based on the elastic theory and stress function method. Moreover, a novel anthropometric method based on image recognition algorithms that systematically measures and evaluates tissue deformation while minimizing the impact of the effects of motion is proposed. The mechanical properties of five leggings samples are tested by using the Instron 4,411 and KES-FB1 systems to determine the uniaxial tension and pure shear.

**Results:**

The predicted results are then compared with the experimental results, which shows that they are in good agreement, with deviations within 1.15 mm for the static condition and 2.36 mm for the dynamic condition, thus validating the proposed novel method.

**Discussion:**

This anthropometric approach is an invaluable tool for evaluating tissue deformation patterns, thus providing key insights for sportswear designers to optimize garment performance and design.

## 1 Introduction

Sports leggings are garments that are commonly used to enhance postural stability and improve gait, thus providing users with a sense of support (Laing and Sleivert, 2002; Nyland et al., 2020). These garments can effectively prevent the accumulation of lactic acid which causes cramps, promote the decomposition of lactic acid to relieve muscle soreness, improve blood circulation, and ease pain after exercise (Attard and Rithalia, 2004; Rennie et al., 2000; Nicholson et al., 2001; Matthews et al., 2009). Despite these benefits, users and athletes do not have the tendency to use sports leggings, which leads to discomfort or soreness due to the high pressure exerted onto the tissues and subsequent tissue deformation (Laing and Sleivert, 2002; Groppo, 2021). Therefore, accurately evaluating and predicting tissue deformation are crucial for optimizing material selection and design for leggings (Barhoumi et al., 2021).

A number of methods have been used to determine the biomechanical response of tissues such as stress, interface pressure, and deformation when subjected to simulated garments. These methods include using Laplace’s law, pressure sensor measurements, finite element (FE) modelling, machine learning, and body scanning (Liang et al., 2019; Ye et al., 2022; Shi et al., 2024; Pei et al., 2021; Yu et al., 2012; Xu et al., 2025). Laplace’s law is often applied to analyse the interface response between garments and the human body (Basford, 2002; Barhoumi et al., 2019). However, it falls short in predicting soft tissue deformation due to garment pressure, as it cannot be used to visualize or adequately predict the mechanical deformation of garments for design optimization.

Pressure sensors are commonly used to measure the pressure at the interface between garments and the human body. However, they cannot detect tissue deformation (Zhao S. et al., 2020). Machine learning is also commonly applied to predict the biomechanical behavior of the human body when wearing compression garments. Xu et al. developed an innovative deep learning model to predict the real-time inversion of ligament loading force-fatigue failure states and estimate fatigue life. Nevertheless, This approach lacks integration with classical mechanical models (e.g., hyperelastic constitutive models), resulting in biased prediction results (Xu et al., 2025). FE modelling is a more comprehensive approach, which can be used to predict interface pressure, tissue stress, and deformation. For instance, Zhao L. et al. (2020) used an FE model to simulate the distribution of pressure on the skin of the upper limbs with the use of elastic sleeves, while Ye et al. (2023) predicted the interface pressure between the lower limbs and compression stockings to understand leg tissue deformation. Shi et al. (2024) estimated the amount of tissue deformation and pressure based on tissue stiffness by using the Hertzian contact theory, which shows the influence of tissue stiffness on deformation. Despite these advancements, previous studies have primarily focused on static biomechanical responses, with the absence of direct estimation methods to examine dynamic tissue deformation.

Body scanning is a direct and visual means of evaluating tissue deformation, and a better method than using Laplace’s law, pressure sensors, and FE models (Greenleaf et al., 2003; Mountney et al., 2010; Palanca et al., 2015; Santesteban et al., 2020). Choi and Hong (2015) investigated the skin length deformation of the lower body during knee joint flexion, and applied the findings to sportswear design. They emphasized the importance of accurately mapping skin deformation to optimize seam locations in compression leggings, although their study only addressed surface deformation, thus neglecting the displacement of the deeper tissues. Liu et al. (2024) proposed an automatic approach by using ultra-dense motion capture and body imaging to analyze the dynamic behavior of the breast surface, thus advancing studies in breast anthropometry and biomechanics. Despite these advancements, limitations remain in evaluating dynamic tissue deformation exerted by compression garments. Misalignment of the cross-section centroids of the leg with and without leggings introduces inaccuracies in the evaluation of tissue deformation.

To address these limitations, this study proposes a novel anthropometric method that uses advanced body scanning technology combined with continuum mechanics solutions to accurately estimate tissue deformation from the use of sports leggings. By employing image recognition algorithms, this method overcomes the challenges of traditional body scanning where displacement from movement affects measurement accuracy. With deviations controlled to within 1.15 mm (static condition) and 2.36 mm (dynamic condition), this model provides crucial insights into the interaction between leggings and the body tissues, which guide the strategic placement of support and stretch zones to optimize fit, wear comfort, and athletic performance. The proposed framework not only advances biomechanical simulation techniques but also provides a practical tool for optimizing sportswear ergonomics - enabling data-driven design of compression garments that enhance athletic performance while preventing musculoskeletal injuries. Furthermore, bridging the gap between complex biomechanical evaluations and practical design applications, this model facilitates a more efficient, data-driven approach to sportswear customization.

## 2 Methodology

### 2.1 Preparation of leggings samples

To evaluate the deformation that results from the pressure exerted onto different areas of the lower limbs, a total of five pairs of sports leggings with different material components, design style, and circumferential dimensions were prepared as the experimental samples. The details are provided in Table 1. All of the leggings were divided into six horizontal sections and labelled sequentially from top to bottom based on biomarkers for the lower body, as shown in Figure 2. The circumferences of the leggings at these horizontal lines were measured, and the values are listed in Table 1. The six sections are defined as follows: Section 1 corresponds to the high hip circumference, Section 2 to the low hip circumference, Section 3 to the mid-thigh circumference, Section 4 to the thigh circumference, Section 5 to the knee circumference, and Section 6 to the calf circumference. The size of each section is as follows: Section 1: 75 mm, Section 2: 90 mm, Section 3: 95 mm, Section 4: 185 mm, Section 5: 100 mm, and Section 6: 195 mm. The sports leggings are assumed to have the same material properties and cut into dimensions of 7.5 cm × 7.5 cm for testing and determining the material properties.

**TABLE 1:** Material composition and design details of leggings samples.

Leggings sample	Contents	Style	Circumferences	Image
1	Nylon:72% Elastane: 28%	Light support, hip-lifting, knee splice mesh, body-wrapping fit	Section 1: 765 mm Section 2: 830 mm Section 3: 424 mm Section 4: 310 mm Section 5: 280 mm Section 6: 220 mm	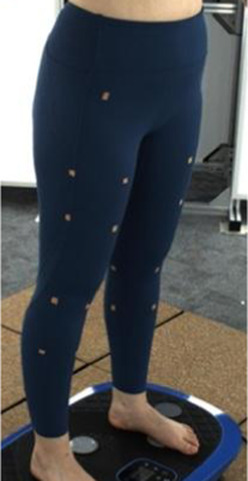
2	Nylon:79% Elastane: 21%	High degree of support, cross-wrap thigh and calf muscle design, hip-lifting	Section 1: 740 mm Section 2: 790 mm Section 3: 490 mm Section 4: 330 mm Section 5: 300 mm Section 6: 234 mm	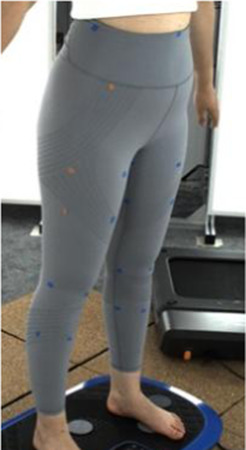
3	Nylon:74% Polyester:19% Elastane: 7%	Seamless knitted construction, cross-wrap design at the waist, different knitted structures to exert different degrees of pressure on body	Section 1: 760 mm Section 2: 840 mm Section 3: 420 mm Section 4: 330 mm Section 5: 300 mm Section 6: 240 mm	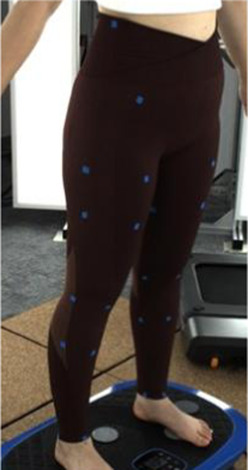
4	Nylon:70% Spandex: 30%	Supported by air-laminated fabric, withstands high impact, one-piece pattern with high-impact pressure resistance	Section 1: 760 mm Section 2: 840 mm Section 3: 450 mm Section 4: 330 mm Section 5: 300 mm Section 6: 230 mm	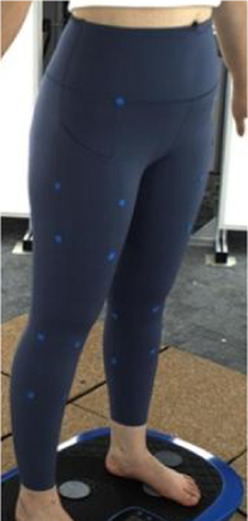
5	Polyester:80% Elastane: 20%	Four-piece regular pattern, does not provide support	Section 1: 800 mm Section 2: 860 mm Section 3: 480 mm Section 4: 340 mm Section 5: 290 mm Section 6: 230 mm	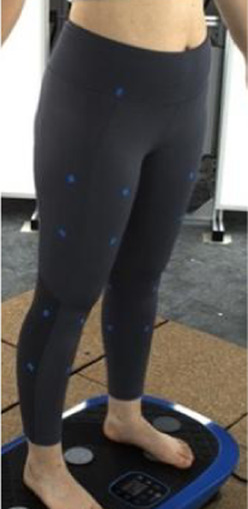

### 2.2 Mechanical properties of leggings samples

Leggings are usually assumed to be an orthotropic lamina as the mechanical properties along their course and wale directions are different. Hooke’s law is applied to characterize the relationships among the elastic modulus (Young’s modulus, Poisson’s ratio, and shear modulus), stress, and strain of the leggings samples (Beer, 2020), which can be expressed as:

$$\left[\begin{array}{l} \varepsilon_{x}\\ \varepsilon_{y}\\ \gamma_{xy} \end{array}\right]=\left[\begin{array}{ccc} S_{11} & S_{12} & S_{16}\\ S_{21} & S_{22} & S_{26}\\ S_{61} & S_{62} & S_{66} \end{array}\right]\left[\begin{array}{l} \sigma_{x}\\ \sigma_{y}\\ \tau_{xy} \end{array}\right]$$ (1)

where *S*_*ij*_ is an orthotropic compliance matrix (OCM) of the leggings samples, *ε*_i_ and *γ*_*ij*_ are the normal and shear strains, respectively, and σi and τij are the normal and shear stresses, respectively. The OCM of the leggings samples can be further expressed with:

$$\left[\begin{array}{l} \varepsilon_{x}\\ \varepsilon_{y}\\ \gamma_{xy} \end{array}\right]=\left[\begin{array}{ccc} \frac{1}{E_{x}} & -\frac{v_{yx}}{E_{y}} & 0\\ -\frac{v_{xy}}{E_{x}} & \frac{1}{E_{y}} & 0\\ 0 & 0 & \frac{1}{G_{xy}} \end{array}\right]\left[\begin{array}{l} \sigma_{x}\\ \sigma_{y}\\ \tau_{xy} \end{array}\right]$$ (2)

where *E*, *v*, and *G* are the Young’s modulus, Poisson’s ratio, and shear modulus of the leggings samples, respectively. Based on Equation 2, the *E*, *v*, and *G* of the leggings samples can be estimated through the measured stress (σ and τ) and strain (ε and γ). Thus, the E, v, and G of the leggings samples are expressed as:

$$E_{x}=\frac{\sigma_{x}}{\varepsilon_{x}};E_{y}=\frac{\sigma_{y}}{\varepsilon_{y}};v_{xy}=-\frac{\varepsilon_{y}E_{x}}{\sigma_{x}};G_{xy}=\frac{\tau_{xy}}{\gamma_{xy}}$$ (3)

Based on Equation 3, the normal stress and strain of the leggings samples were measured by using the Instron 4,411 system (Norwood, MA, USA). The shear stress and strain of the leggings samples were measured by using the Kawabata evaluation system (KES-FB1, Kyoto, Japan). The tested material properties were imported into our proposed analytical model to estimate the tissue deformation.

### 2.3 Analytical model to determine deformation of the leg tissues

To predict the deformation of the tissues of the legs due to the use of the leggings (hereinafter the leggings-leg system), the interface pressure between the leggings and soft tissues of the legs was generated in two ways: i) through the extension of the compression garments (static), and ii) through inertial forces (dynamic). The static tissue deformation was initially predicted by using the Boussinesq solution based on the elastic theory. Then, soft tissue deformation can be estimated based on our predicted interface pressure via a stress function.

The cross-section of the legs is assumed to be a regular circle. Therefore, the Boussinesq solution based on the elastic theory for the leggings-legs system can be obtained with:

$$u_{l}=L-\frac{1}{2\left(1-v\right)}\nabla \left(\Phi +\frac{1}{2}RL\right)$$ (4)

where *Φ* is a stress function, *R* denotes the radius of the cross-section of the leg, ▽ is a noble operator, *u*_*l*_ is the deformation of the legging samples, and *L* is a harmonic function of the stress function.

The solution for Equation 4 of the leggings-legs system can be obtained by using:

$$u_{l}=\frac{\left(3-4v\right)K-C}{R}-\frac{Kz^{2}}{R^{3}}$$ (5)

Considering the axial symmetry of the leggings-legs system, the boundary conditions of this system are expressed by using:

$${\left(\sigma_{r}\right)}_{r=R}=0;\;{\left(\tau_{r\varphi }\right)}_{r=R}=0$$ (6)

Based on the boundary conditions, the relationship between the deformation of the leggings and interface pressure can be expressed by using:

$$u_{l}=\frac{\left(1-v_{xy}^{2}\right)P}{\pi E_{x}\frac{R}{2}}$$ (7)

where *v*_*xy*_ and *E*_*x*_ are the Poisson’s ratio and Young’s modulus of the tested leggings samples in the course direction, respectively, and *P* is the pressure at the interface between the leg and leggings.

**FIGURE 1:**
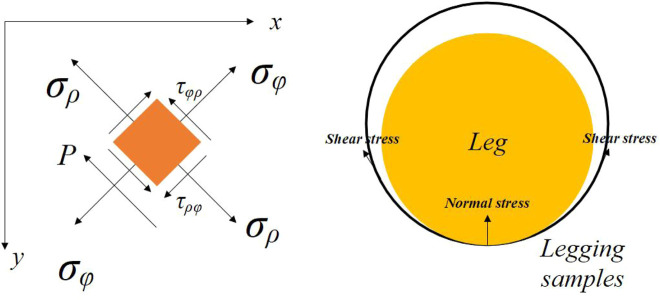
Mechanical mechanisms of the leggings-legs system.

Based on the predicted pressure at the interface between the leg and leggings, the tissue deformation can be estimated by using a stress function. The mechanical balance equation of the leggings-legs is formulated with cylindrical coordinates as follows (see Equation 8):

$$\begin{array}{l} \frac{\partial \sigma_{\rho }}{\partial \rho }+\frac{1}{\rho }\frac{\partial \tau_{\varphi \rho }}{\partial \varphi }+\frac{\sigma_{\rho }-\sigma_{\varphi }}{\rho }+f_{\rho }=0\\ \frac{1}{\rho }\frac{\partial \sigma_{\varphi }}{\partial \varphi }+\frac{\partial \tau_{\rho \varphi }}{\partial \rho }+\frac{2\tau_{\rho \varphi }}{\rho }+f_{\varphi }=0 \end{array}$$ (8)

where *σ*_*ρ*_ and *σ*_*φ*_ are the normal stresses of the leggings fabric along the radial and circumferential directions (Figure 1). The relationship between the stress and strain is obtained by using Hooke’s law:

$$\varepsilon_{\rho }=\frac{1}{E_{x}}\left(\sigma_{\rho }-v_{xy}\sigma_{\varphi }\right);\;\varepsilon_{\varphi }=\frac{1}{E_{y}}\left(\sigma_{\varphi }-v_{xy}\sigma_{\rho }\right);\;\gamma_{\rho \varphi }=\frac{1}{G_{xy}}\tau_{\rho \varphi }$$ (9)

where *E*_*x*_, *E*_*y*_, and *v*_*xy*_ are the Young’s modulus and Poisson’s ratio in the warp and weft directions of the leggings samples, and *G*_*xy*_ is the shear modulus of the leggings samples.

Based on the stress function (*Φ*), the stress components of the legs-leggings system can be derived by using:

$$\rho^{4}\frac{d^{4}\Phi }{d\rho^{4}}+2\rho^{3}\frac{d^{3}\Phi }{d\rho^{3}}-\rho^{2}\frac{d^{2}\Phi }{d\rho^{2}}+\rho \frac{d\Phi }{d\rho }=0$$ (10)

The stress function can be solved by using the Euler function as follows:

$$\Phi =A\ln\rho +B\rho^{2}\ln\rho +C\rho^{2}+D$$ (11)

where *A*, *B*, *C*, and *D* are the undetermined coefficients. Taking into consideration the axial symmetry of the leggings-legs system, the stress component of the leggings fabric can be obtained by using:

$$\sigma_{\rho }=\frac{A}{r^{2}}+2C;\;\sigma_{\varphi }=-\frac{A}{r^{2}}+2C;\;\tau_{r\varphi }=0$$ (12)

The boundary conditions of the leggings fabric during interaction with the tissues can be obtained as follows:

$${\left(\sigma_{r}\right)}_{r=a}=-P;\;{\left(\sigma_{r}\right)}_{r=b}=0;\;{\left(\tau_{r\varphi }\right)}_{r=a}=0;\;{\left(\tau_{r\varphi }\right)}_{r=b}=0$$ (13)

Based on the boundary conditions of the leggings fabric, the tissue deformation is obtained with:

$$u_{s}=\frac{\left(1-v\right)\mathrm{PR}}{E_{s}}$$ (14)

where *u*_*s*_ is the soft tissue deformation and *E*_*s*_ is the Young’s modulus of the soft tissues. A Neo-Hookean nonlinear strain energy density function was governed to characterize the non-linear behaviour of the soft tissue, which can be expressed as:

$$W=C_{1}\left({\bar{I}}_{1}-3\right)+\frac{1}{D_{1}}{\left(J-3\right)}^{2}$$ (15)

where *W* is the strain energy density, and *C*_1_ and *D*_1_ are the constitutive parameters of the Neo-Hookean hyperplastic model, which have values that are approximately 5,000 Pa and 1.4 × 10–^7^ Pa^−1^, respectively (Zhao L. et al., 2020). Moreover, *I*_1_ is the first invariant of the Cauchy-Green deformation tensor and *J* is the volume ratio. The relationship among the constitutive parameters, Young’s modulus, and Poisson’s ratio of the soft tissues can be expressed as:

$$C_{1}=\frac{E_{s}}{4\left(1+2v_{s}\right)};D_{1}=\frac{3E_{s}}{2\left(1-2v_{s}\right)}$$ (16)

where *μ* and *λ* are the shear modulus and bulk modulus, respectively. Thus, the tissue deformation is obtained with:

$$u_{s}=\frac{\left(D_{1}+3C_{1}\right)\left(1-\frac{D-6C_{1}}{6C_{1}+2D_{1}}\right)\mathrm{PR}}{6C_{1}D_{1}}$$ (17)

Therefore, Equation 17 is used to calculate the static tissue deformation of the six horizontal sections along the cross-section of the five different samples.

The dynamic tissue deformation is predicted by using a dynamic function, which can be expressed as:

$$\begin{array}{l} u_{ds}=\frac{\left(D_{1}+3C_{1}\right)\left(1-\frac{D-6C_{1}}{6C_{1}+2D_{1}}\right)\left(P+F\right)R}{6C_{1}D_{1}}\\ mu_{dl}^{\prime \prime }+k_{dl}^{\prime }=F \end{array}$$ (18)

where *m* is the legging mass, *u*_*dl*_ and *u*_*ds*_ are the tissue dynamic displacement, and *F* denotes the inertial force. The Runge-Kutta methods are used to solve Equation 18 by using MATLAB v2016a software (Mathwork, USA).

### 2.4 Body scanning and image collection

**FIGURE 2:**
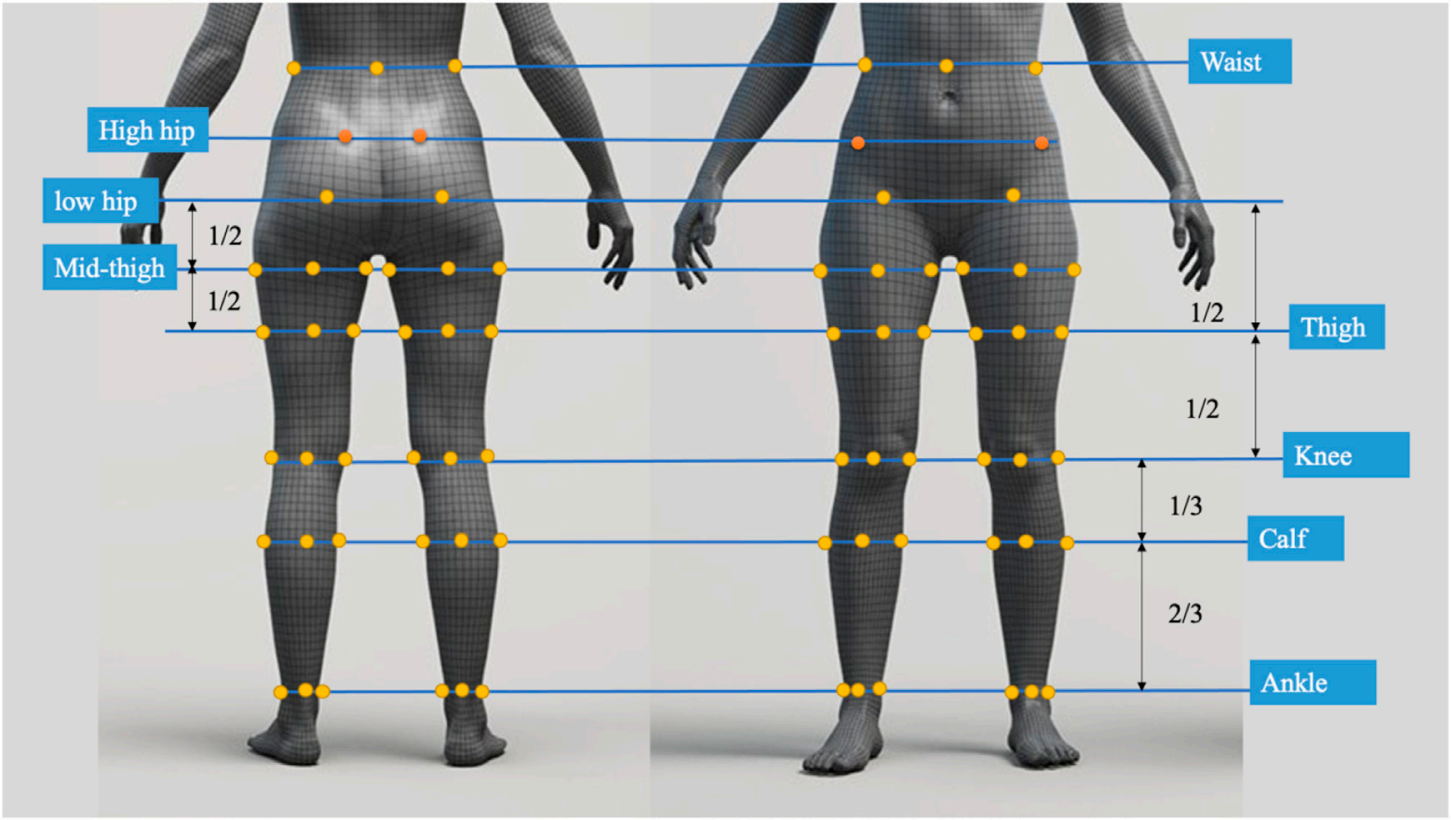
Landmark locations on lower limbs.

In Section 2.3, an analytical model that predicts tissue deformation is described which takes into consideration the average value of the leg curvature. However, it is very difficult to measure the curvature of the cross-section of the leg because of the complex 3D contours. To obtain the measurements, body scanning along with image collection is proposed in this study. Therefore, a subject with a body mass index (BMI) over 24 was recruited for this part of the study. This BMI falls within the “overweight” category according to WHO Asian classification standards and represents individuals with relatively higher adiposity. Such body types are more likely to exhibit pronounced soft tissue deformation and complex garment-body contact behavior. This subject was informed of the experimental procedures. She agreed to participate and signed an informed consent form. Human ethics approval was granted by the Human Subjects Ethics Application Review System of the university of the first author.An advanced body scanning system (3dMD LLC, USA) was used to scan the contours of the subject. Before the experiment began, landmarks based on the anatomy and morphology of the lower limbs were marked on the surface of the lower body, as shown in Figure 2.

**FIGURE 3:**
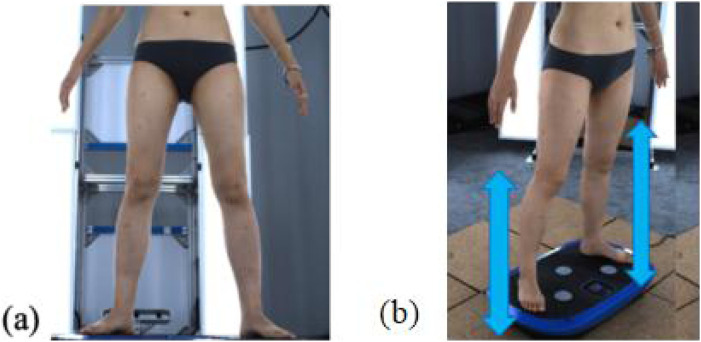
4D lower body scanning: **(a)** static standard A-pose, and **(b)** whole-body vibration with vertical vibration machine.

**FIGURE 4:**
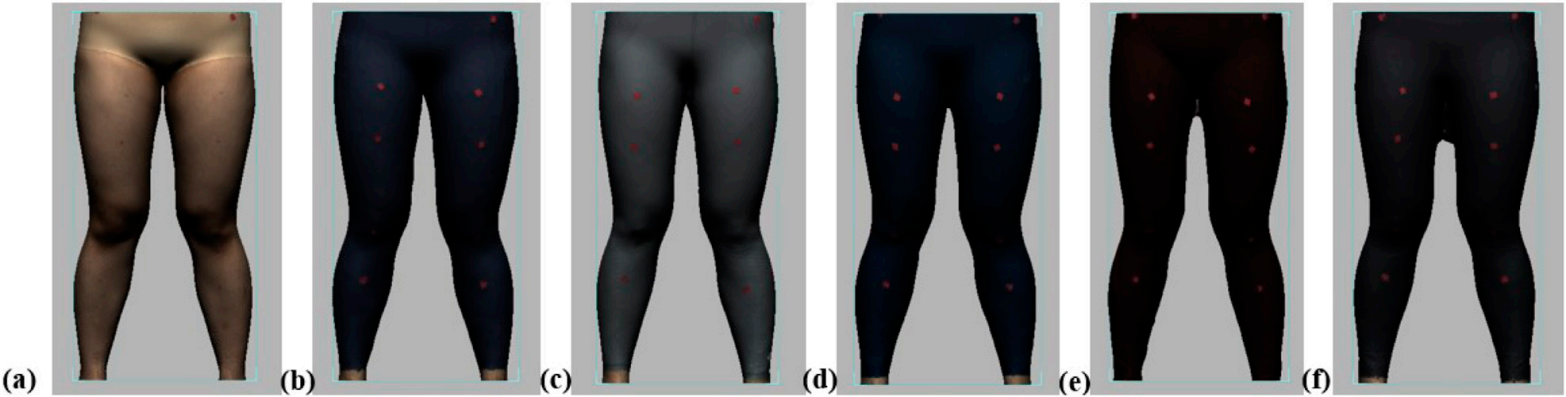
Scanning conditions: **(a)** thong only, and wearing **(b)** Leggings Type 1, **(c)** Leggings Type 2, **(d)** Leggings Type 3, **(e)** Leggings Type 4, and **(f)** Leggings Type 5.

**FIGURE 5:**
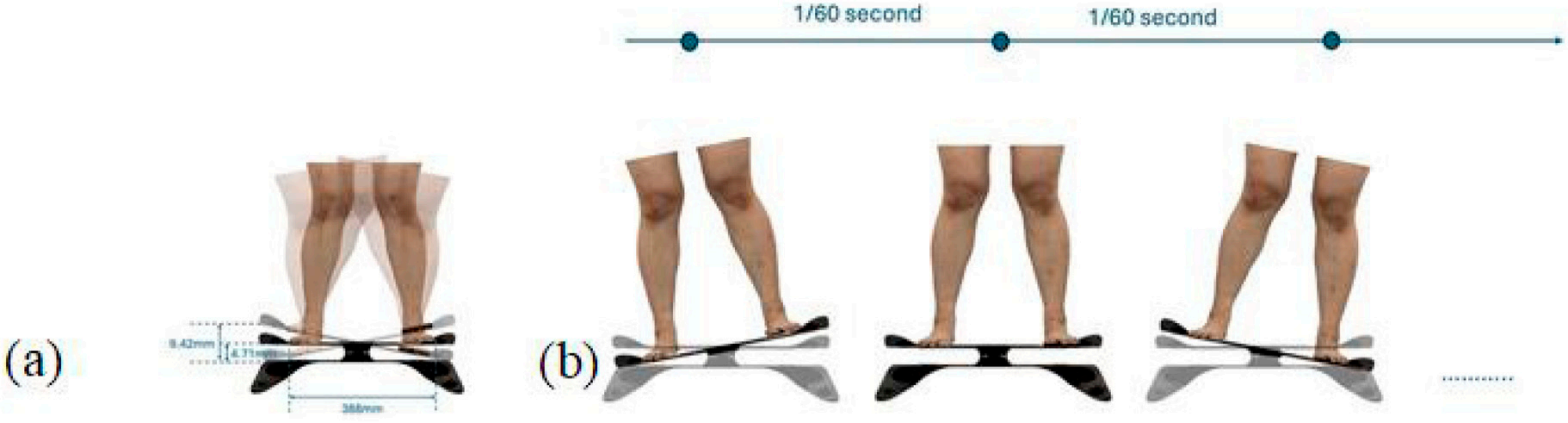
Selected frames during vibration cycle: **(a)** vertical displacement, and **(b)** time interval.

Throughout the experiment, the scanning consisted of static 3D scanning in the standard A-pose, as shown in Figures 3a, 4d dynamic scanning which was recorded at 120 frames per second while standing on a whole-body vertical vibration machine at a vibration frequency of 60 Hz, as shown in Figure 3b. While a vibration frequency at 1 Hz simulates the running cadence, higher muscle activation and muscle stiffness can be achieved at higher vibration frequencies (Krol et al., 2011), thereby better simulating the dynamic mechanical behavior of soft tissues under high-impact conditions. The selected frequency of 60 Hz corresponds to the upper range of soft tissue vibrations observed during running and jumping (typically 10–60 Hz), and thus serves as an effective approximation for evaluating the biomechanical response of the body under intense dynamic loading. The scans were performed under six conditions: thong only (Figure 4a), and wearing Leggings Types 1, 2, 3, 4, and 5 (Figures 4b–f). To establish a baseline for tissue deformation, a “thong only” condition—providing negligible compression—implemented as the control. All deformation values reported in this study represent net deformation, calculated by subtracting the tissue deformation measured under the thong-only condition from that measured under each compression garment condition. This approach isolates the garment-induced deformation from natural body movement or baseline soft tissue behavior, thereby enhancing the validity of the comparisons across different leggings. At least 5 consecutive cycles were recorded for each running scan, and the cycle with the best scan coverage was selected for further analysis. To gain a better understanding of the lower body deformation, the static standard A-pose scans and dynamic scans during the vertical vibration of the entire body were extracted separately. For the static scans, landmarks of the lower body that correspond to the newly created landmarks on the 3D images were used. For the dynamic scans, two critical frames were selected from each vibration cycle—the frames with the highest and lowest vibration levels—as shown in Figure 5. The vertical displacement between these two frames is 9.42 mm as shown in Figure 5a, and the time interval is 1/30 s, as shown in Figure 5b. Similarly, these key frames established the new landmarks that correspond with the experimental landmarks.

**FIGURE 6:**
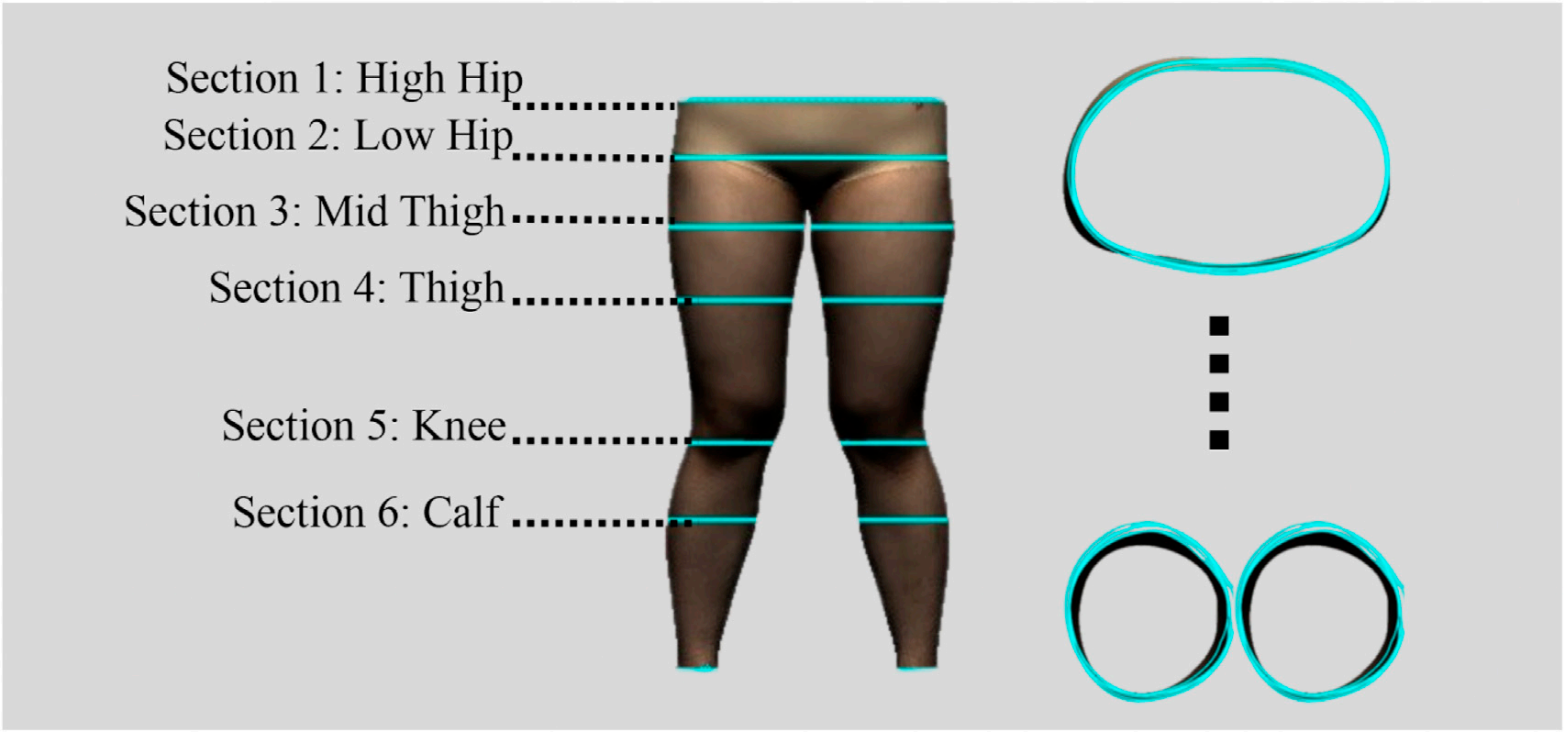
Radial segmentation of lower body.

The radial curves were segmented by using these new landmarks. All of the landmarks were connected horizontally by using Geomagic Design X 2020 software (3D Systems, Korea). Cross-sections of the hip and the left and right legs were recorded and measured separately. Horizontal lines were drawn at the hips, the high points of the left and right thighs, left and right knees, left and right calves, and lower parts of the left and right calves, respectively, as shown in Figure 6. Radial segmentation was then carried out. The scanned STL files were further imported into image recognition software and an image recognition code was used to estimate the deformation of the leg tissues, before and after wearing the sports leggings prototypes, for all of the static and dynamic trials.

### 2.5 Measurement of tissue deformation of leggings-legs system through image recognition and statistical analysis

**FIGURE 7:**
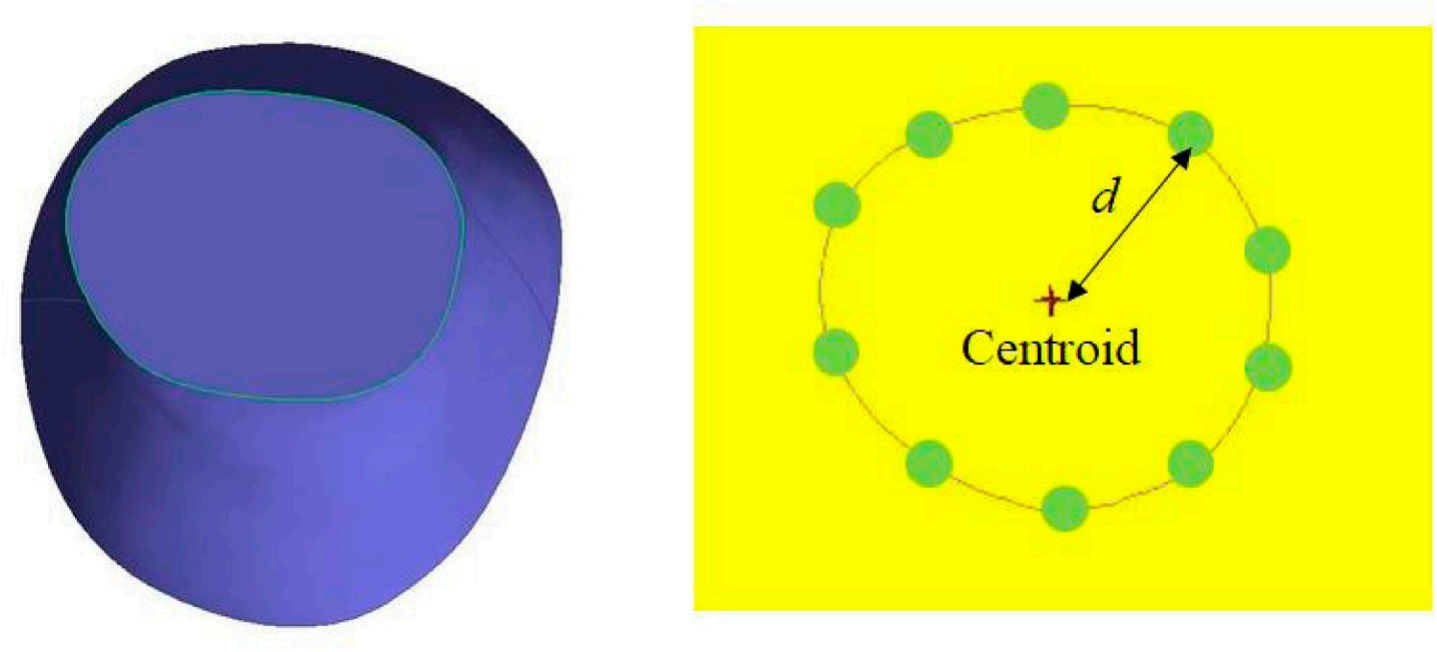
Centroid and curvature radius of leg.

To determine tissue deformation based on the scanned data along with the complex contours of the cross-section of the leg, a novel imaging recognition method that can identify cross-sectional shapes is used in this study. The original scanned files of the legs were in STL format. These files were subsequently imported into CAD software and the images of the six different conditions were extracted. These images were then imported into MATLAB software v 2016a, which uses an image recognition code. The contours of the leg were marked in yellow and the code obtained the *x*- and *y*-coordinates of the yellow section based on an RGB color model with a threshold value of 1 (8-bit range: 0–255). The algorithm demonstrated high repeatability, as the centroid coordinates remained consistent across repeated computations. The centroids of the cross-section of the leg with the five leggings samples and without the use of the leggings were determined based on the average *x*- and *y*-coordinates, respectively, of all the points that were marked in red (Figure 7). The equation for determining the centroid is:

$$\begin{array}{l} x_{o}=\frac{\sum x_{i}}{n}\\ y_{o}=\frac{\sum y_{i}}{n} \end{array}$$ (19)

where *x*_*i*_ is the *x*-coordinate and, *y*_*i*_ the *y*-coordinate in the Cartesian coordinate system, and *n* denotes the points extracted of the soft tissue contours based on the image recognition algorithm.

Based on the centroid of the cross-section of the leg, the distances between the contours and centroid are determined by using:

$$d=\sqrt{{\left(x_{o}-x_{i}\right)}^{2}+{\left(y_{o}-y_{i}\right)}^{2}}$$ (20)

The tissue deformation was determined based on the differences between the leg curvature radius with and without the leggings. The average amount of tissue deformation of the six conditions for each sample leggings along the cross section and and left/right legs was recorded and compared with the predicted values to estimate the deviation values and significant differences by using an analysis of variance (ANOVA).

Prior to ANOVA, all tissue deformation datasets underwent normality verification via Shapiro-Wilk testing. The confirmation of normal distribution in all sample groups (p > 0.05) validated the parametric assumptions for ANOVA. Subsequently, we conducted one-way ANOVA with F-tests to evaluate differences between predicted and measured deformation data, with between-group comparisons performed for different garment types, anatomical sections, and left/right leg positioning.

## 3 Results

### 3.1 Mechanical properties of leggings samples

To evaluate the physical properties of the materials used in all of the samples, the mechanical properties were tested in both the warp and weft directions. A linear regression analysis of the loading and strain of the leggings samples showed that the Pearson’s correlation coefficient is approximately 0.99, which means that the fabrics can be assumed to be a linear elastic material. Specifically, the Young’s modulus in the warp direction corresponds to the *E*_*x*_ values along the course direction of the fabric, while that in the weft direction is represented by the *E*_*y*_ values along the wale direction. The Poisson’s ratio and shear modulus of the plane along the length of the course and wale directions are represented by the *v*_*xy*_ and *G*_*xy*_ values, respectively. Table 2 lists the physical and mechanical values of the leggings fabrics examined in this study. The results reveal differences in the Young’s modulus (*E*) among the different leggings. Specifically, the *E*_*x*_ values for Leggings Types 1, 2, and 3 are relatively consistent. Moreover, the *E*_*x*_ values for Leggings Types 1 and 2 are similar to the *E*_*y*_ values. However, Leggings Type 3 exhibits a significantly higher *E*_*y*_ value compared to *E*_*x*_, thus indicating enhanced shape retention during longitudinal stretching. Notably, Leggings Type 4 has the highest *E*, while Leggings Type 5 has the lowest. This suggests that the former has superior shape retention when stretched and provides more consistent pressure onto the body thus resulting in a higher degree of deformation. In contrast, Leggings Type 5 has the lowest shape retention, causes less deformation, and exerts the least amount of pressure. The Poisson’s ratio of the five leggings samples is approximately 0.15. The shear modulus is approximately 650 Pa for all of the leggings samples.

**TABLE 2:** Mechanical and physical values of leggings fabrics.

Leggings sample	*E*_*x*_ (Pa)	*E*_*y*_ (Pa)	*G*_*xy*_ (Pa)	*v*_*xy*_
1	2.23E+03	2.46E+03	657.40	0.13
2	2.67E+03	2.25E+03	660.09	0.12
3	2.82E+03	4.01E+03	647.52	0.12
4	4.35E+03	3.79E+03	661.26	0.16
5	1.72E+02	2.14E+03	649.78	0.13

### 3.2 Calculations of tissue deformation exerted by leggings on lower limbs

**FIGURE 8:**
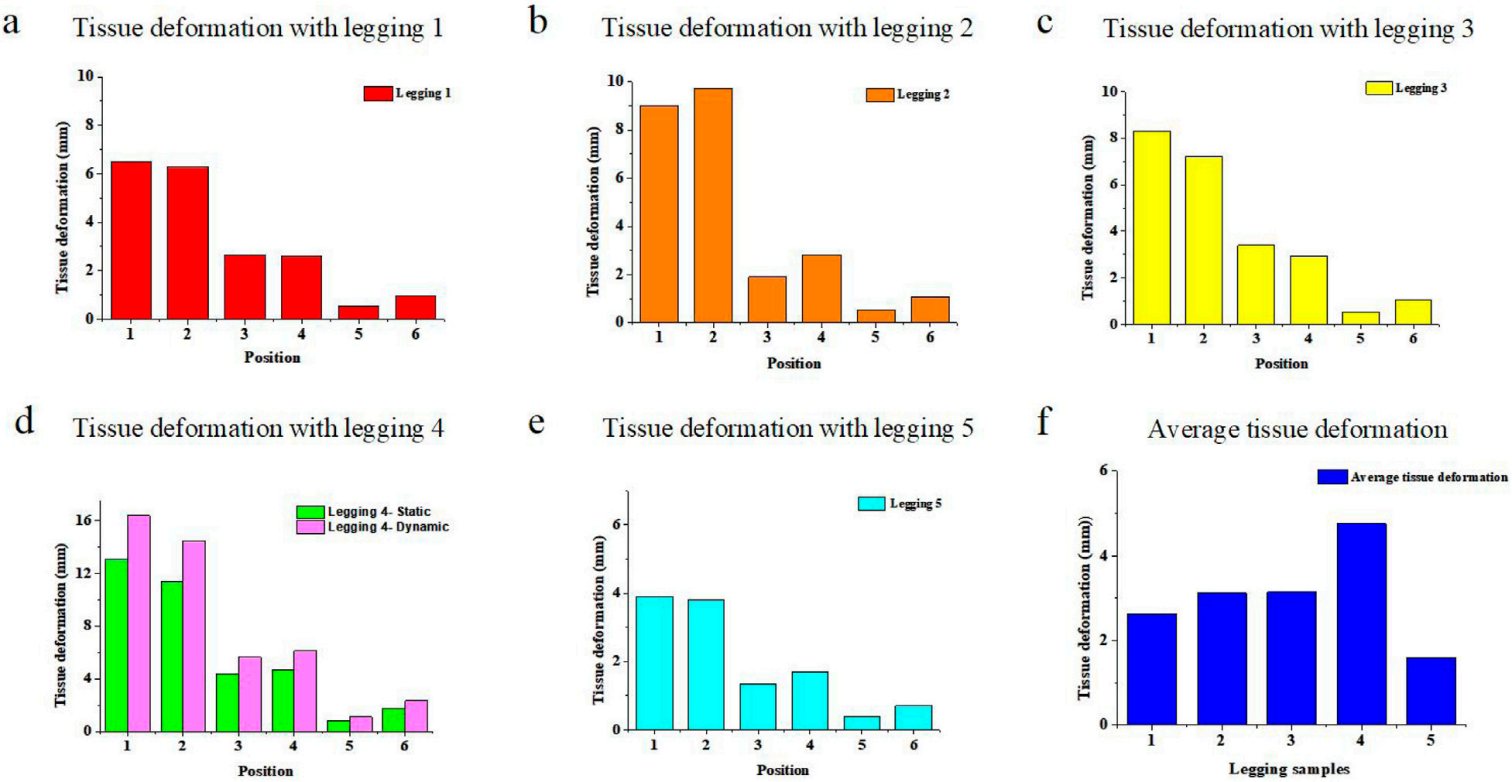
Plotted tissue deformation for six sections with Leggings Types **(a)** 1, **(b)** 2, **(c)** 3, **(d)** 4 and **(e)** 5, and **(f)** average tissue deformation in the six sections.

The plots in Figure 8 show the predicted amount of deformation of the soft tissues resultant of using the leggings which exert pressure on the legs and along the legs. The average calculated amount of deformation of the six sections for Leggings Types 1 to 5 is approximately 2.63 mm, 3.12 mm, 3.14 mm, 4.75 mm, and 1.60 mm, respectively. It can be seen that Leggings Type 4 causes the most deformation, while Leggings Type 5 causes the least deformation, which indicates that a higher Young’s modulus causes more deformation, and correspondingly, a lower Young’s modulus causes less deformation with Leggings Type 5 because it is larger in size (BS ISO 5971:2017 Size designation of clothes-tights). Therefore, the material properties of the leggings samples influence tissue deformation.

Most of the deformation was found along the hip section and gradually decreased towards the ankle. For instance, the calculated tissue deformation in Section 1 is approximately 6.4 mm, and that in Section 4 is approximately 2.4 mm with Leggings Type 1. In Section 6, the calculated tissue deformation is reduced to 0.95 mm with Leggings Type 1, thus indicating that tissue deformation is affected by the stretching of the leggings along the leg. The leggings are stretched approximately 150, 170, and 180 mm along the cross view. Therefore, leggings with more stretch cause more pressure and tissue deformation. Leggings size selection is also a crucial factor in determining tissue deformation. In summary, based on the developed analytical model, tissue deformation is affected by both the leggings materials and leggings size.

In the cross-sectional view, the posterior of the lower limbs has a lower curvature radius at Section 4 or 8.0 mm with Leggings Type 4, versus the other sections, such as the anterior of the large limbs, which is 6.5 mm. Furthermore, the tissue curvature radius at the lateral and medial positions is 7.4 mm and 7.2 mm, respectively. Based on Equation 17, the calculated tissue deformation is 5.23 mm, 4.31 mm, 4.7 mm, and 4.88 mm at the posterior, anterior, lateral, and medial positions, respectively. The differences in deformation at the cross-sections are caused by the differences in curvature radius.

Figure 8d shows the dynamic tissue deformation with Legging Type 4 calculated based on Equation 18. The average dynamic tissue deformation is 16.37 mm, 14.47 mm, 9.70 mm, 8.17 mm, 6.06 mm, and 6.03 mm for the six sections in one cycle, respectively. It can also be seen that the dynamic tissue deformation values are slightly higher than the static tissue deformation values caused by inertial force.

### 3.3 Measured tissue deformation exerted by sports leggings on the lower limbs

**FIGURE 9:**
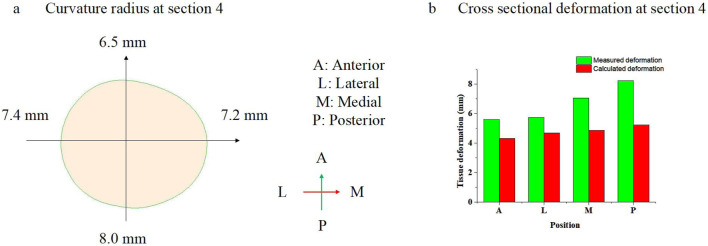
**(a)** Curvature radius of leg in Section 4, and **(b)** cross-sectional deformation in Section 4.

**FIGURE 10:**
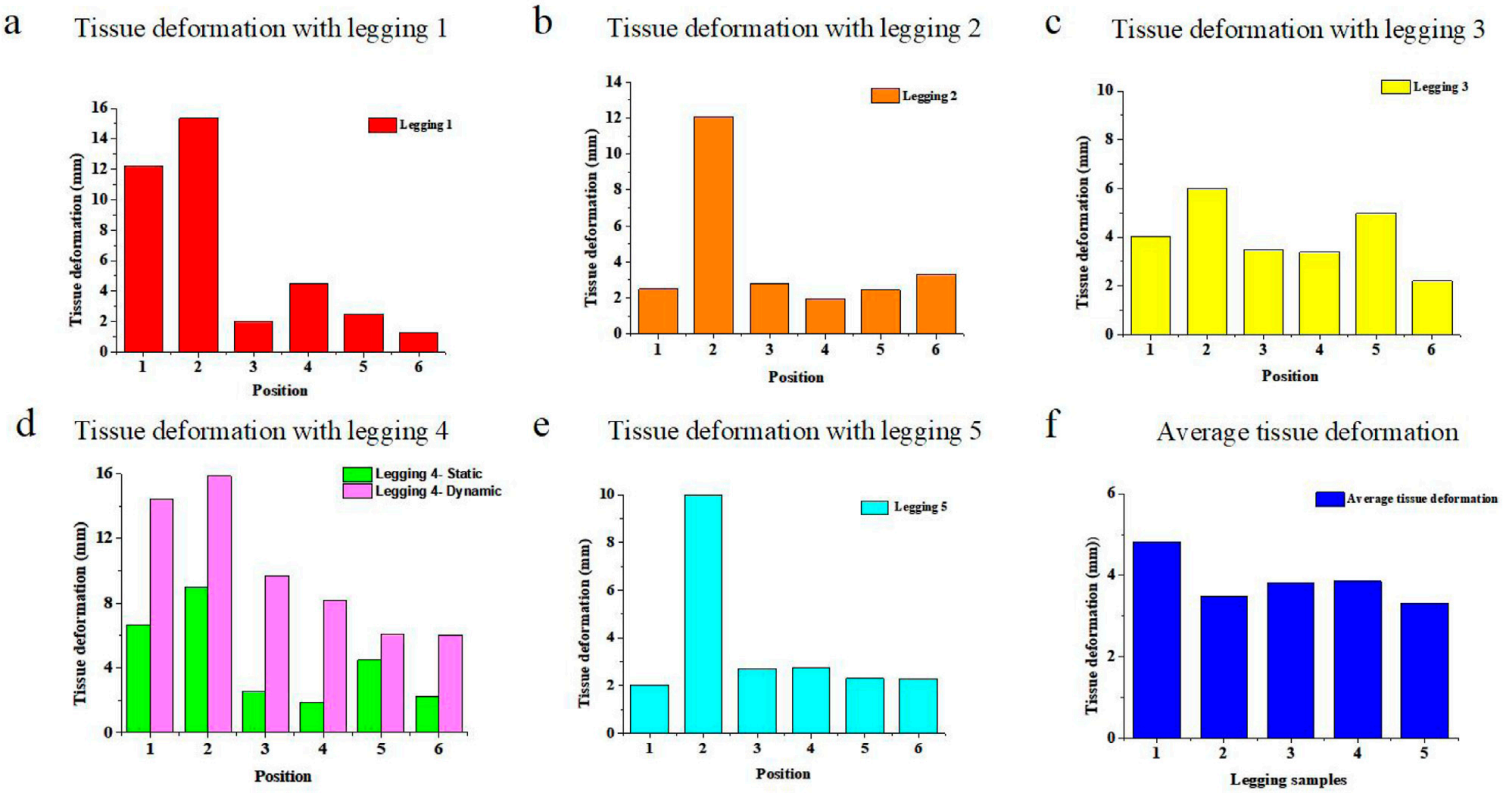
Measured tissue deformation at six sections for Leggings Types **(a)** 1, **(b)** 2, **(c)** 3, **(d)** 4 and **(e)** 5, and **(f)** average tissue deformation of the six sections.

The graphs in Figures 9, 10 show the measured static tissue deformation based on body scanning. The proposed analytical model is used to predict the average tissue deformation in Section 3.2. However, the cross-section of the leg has 3D contours, which makes it difficult to measure, and thus body scanning was subsequently conducted. It was found that the average tissue deformation of the six sections is 4.82 mm, 3.49 mm, 3.82 mm, 3.85 mm, and 3.31 mm for Leggings Types 1 to 5, respectively, which is similar to the predicted results. However, in this case, Leggings Type 1 causes the most deformation. The experimentally measured tissue deformation along the longitudinal plane is also similar to the predicted results, in which there is more deformation in Section 1 and Section 2.

In the cross-sectional view, the posterior of the lower limbs shows more deformation at Section 4 or 8.24 mm with Leggings Type 4, versus the other sections, such as the anterior of the lower limbs, which is 5.60 mm, lateral position of the lower limbs, which is 5.73 mm, and medial position of the lower limbs, which is 7.07 mm. Compared with the modeled results, the deformation differences around the cross-section are also caused by the difference in the curvature radius of the lower limb.

We also examined the measured dynamic tissue deformation with Leggings Type 4 based on the body scanning results. The results showed that the largest average tissue deformation value of the six sections is 14.41 mm, 15.87 mm, 9.70 mm, 8.17 mm, 6.06 mm, and 6.03 mm, respectively. Similarity, the lowest average tissue deformation of six sections are 15.42 mm, 16.02 mm, 9.67 mm, 8.43 mm, 6.25 mm, and 5.87 mm, respectively. The measured values of the dynamic tissue deformation are in agreement with the predicted results.

### 3.4 Analysis of predicted vs. measured tissue deformation

**FIGURE 11:**
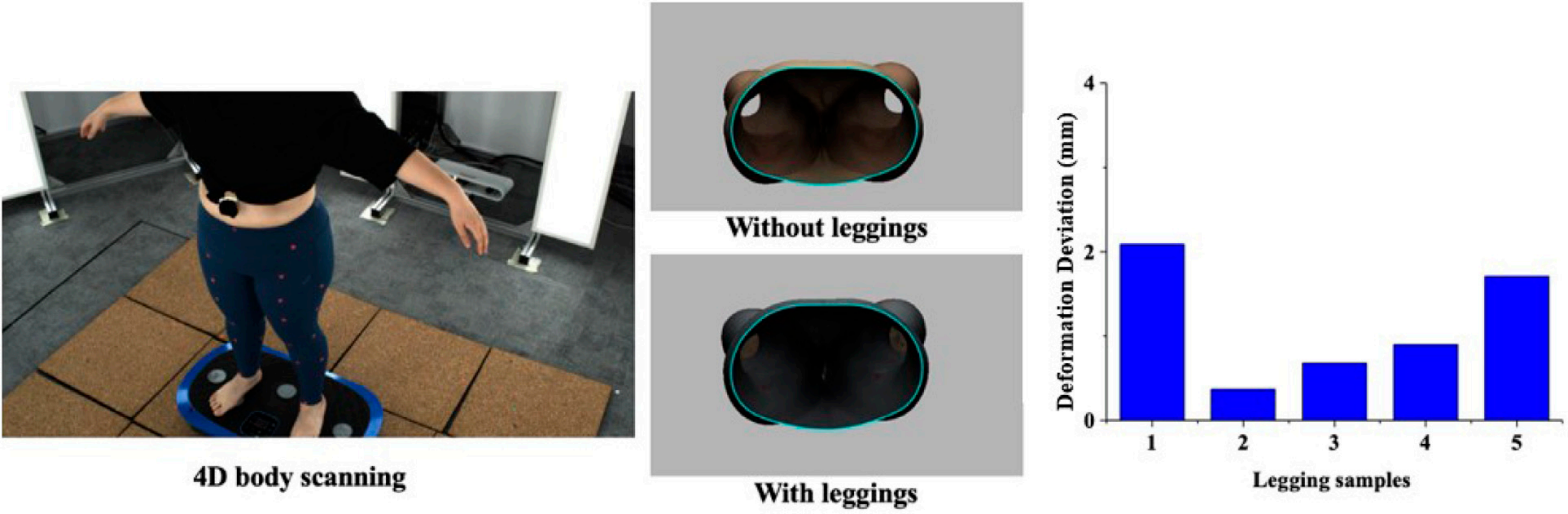
Difference between predicted and measured tissue deformation.

Figure 11 shows the difference between the predicted deformation values and measured average amount of deformation. The results indicate that the predicted and measured data are generally in agreement with a deviation of 1.15 mm (static condition) and 2.36 mm (dynamic condition). However, there is a notable difference between the two types of measurements in Section 1 and Section 2. This discrepancy can be attributed to the shape of the hip area, which is more curved where Section 1 and Section 2 are found, compared to the more circular shape of the leg, which has less curvature. Conversely, the differences between the predicted and measured data in Sections 3 through 6 are relatively smaller, as the cross-sectional shape of the leg is closer to that of a regular circle. Moreover, the predicted and measured values differ between the left and right legs, which can be explained by the inherent asymmetry of the human body. This finding suggests that anthropometric measurements should be conducted separately for each side of the body. Finally, the variation in deformation caused by the forces exerted by the leggings of different designs across different body regions leads to differences in the predicted and measured values for each design.

Table 3 shows the statistical significance of the differences among the five leggings samples by comparing the left and right legs, as well as differences across each section. The results show that there is no significant difference (p > 0.05) in tissue deformation between the left and right legs with Legging Types 2 to 5, thus indicating that both sides of the body are relatively symmetrical. However, there are significant differences (p < 0.005) in tissue deformation from Sections 1–6 with the exception of Leggings Type 3, which suggests that the deformation is related to the size of these sections.

**TABLE 3:** Statistical significance of differences between left and right legs and tissue deformation.

Leggings	Left and right legs	Section
1	p < 0.05*	p < 0.005*
2	p > 0.05	p < 0.005*
3	p > 0.05	p > 0.05
4	p > 0.05	p < 0.005*
5	p > 0.05	p < 0.005*

Note: p < 0.05: significance at the 5% level (statistically significant) and p < 0.005: significance at the .05% level (highly statistically significant).

## 4 Discussion

A number of studies have explored the different methodologies for evaluating and analysing soft tissue deformations of the surface of the body. Notably, the measurement of soft tissue deformation by using body scanning has gained significant attention in the fields of sports science (Spahiu and Kyosev, 2023; Yang et al., 2024; Alemany et al., 2023; Klepser and Morlock, 2020; Sitnik et al., 2019). Body scanning and analysis are invaluable in fields like sports science, where understanding tissue movement is crucial for designing effective interventions and optimizing athletic performance. While body scanning provides a direct and comprehensive observation of how tissues deform and interact dynamically with the use of compression garments (Apeagyei, 2010), the current methods cannot track the patterns of deformation.

To address the issue, our proposed novel anthropometric method significantly enhances the design process of leggings by providing accurate and dynamic insights into soft tissue deformation. This method offers several advantages that lead to more effective design decisions. First, traditional analyses of soft tissue deformation with body scanning techniques often face challenges with displacement due to movement under various scanning conditions, thus resulting in inaccuracies that can affect design decisions. The proposed method in this study minimizes the effects of displacement, thus ensuring accurate measurements of tissue deformation with deviations as low as 1.15 mm (static condition) and 2.36 mm (dynamic condition). This high level of accuracy enables designers to obtain reliable data that genuinely reflects soft tissue deformation with various leggings. Understanding these deformation patterns is crucial for optimizing garment fit and wear comfort. Designers can strategically place support and stretch zones in the leggings to correspond with areas of significant deformation, thus enhancing wear comfort and athletic performance. For instance, incorporating more elastic materials in areas of high deformation can reduce discomfort and improve mobility. This level of accuracy facilitates a more informed selection of materials and garment structures that align closely with the dynamic contours of the body.

Moreover, the integration of advanced image recognition algorithms streamlines the data collection and analysis process. This technological advancement effectively bridges the gap between complex biomechanical analyses and practical garment design, which increases the efficiency of the process. Consequently, this method allows for customization and accommodates a wide range of different body types and movement patterns. By using dynamic anthropometric data, designers can customize leggings, which is particularly important for sportswear, where individual differences can significantly impact athletic performance and wear comfort. Tailoring garments to meet the specific needs of various user groups enhances the overall value and appeal of the product itself.

Our results show that the predicted and measured tissue deformation values are in good agreement. While the deviation is only 1.15 mm for the static condition and 2.36 mm for the dynamic condition, there are reasons for the difference which might be due to several factors: i) the predicted tissue deformation neglects the 3D contours of the legs as the leg is considered to be a circle along the cross-section, and thus curvature variations are considered in the measured tissue deformation; for cases involving highly irregular body morphology, the model can be adapted by incorporating local curvature parameters to replace the simplified radius assumption; ii) the stiffness of the tissues is based on data of previous studies, and some differences in stiffness can be found among the different subjects; iii) bones are not considered in the proposed model, which may somewhat influence the modeled degree of tissue deformation; and iv) the analytical model omits the viscoelastic properties of compression leggings, which can lead to time-dependent changes in skin pressure and tissue deformation—particularly relevant for dynamic scenarios. A deviation of 2 mm between the predicted and measured tissue deformation values indicates that the proposed analytical model and measurement methods have a similar accuracy in determining tissue deformation. Some previous studies have applied the FE model to evaluate tissue deformation with compression garments; for example, Gu et al. (2024) simulated tissue deformation at the knee with knee sleeves and found that the simulated deformation is approximately 0.3 mm. Shi et al. (2024) simulated tissue deformation in the lower limb with compression stockings and found that the average value of the tissue deformation is 3.6 mm. They showed that tissue deformation is influenced by both the tissue and textile properties. Compared to previous studies, our predicted and measured tissue deformation values are in agreement with similar material properties of the textiles used and soft tissues.

Our novel anthropometric method and ultra-dense motion capture (UdMC), proposed by Liu et al. (Choi and Hong, 2015), are advanced techniques for analysing soft tissue deformation, with each addressing specific challenges based on unique methodologies. Our anthropometric method integrates an analytical model based on elastic mechanics, the Boussinseq solution and stress function method, and image recognition based on body scanning data to evaluate tissue deformation with the use of sports leggings, thus effectively minimizing the effects of displacement cause by movement. By incorporating mechanical property testing, this method can accurately predict tissue deformation, validated against body scanning measurements with deviations within 1.15 mm. The results provide actionable insights for optimizing sportswear design by linking material properties to garment fit and performance. In contrast, the UdMC employs thin-plate spline (TPS) interpolation and sparse motion capture landmarks to realize dense tracking at the vertex level, which is optimal for dynamic breast motion analyses. While the UdMC offers unparalleled spatial resolution for dense tracking, it relies on computational validation and is prone to accumulated errors over extended sequences. Both methods have complementary strengths, with our anthropometric method excelling in practical applications for sportswear design and UdMC in high-resolution motion tracking.

This anthropometric method proposed in this study not only advances the evaluation of soft tissue deformation but also offers substantial practical implications for ergonomic garment design and customized wearable devices. The precision and dynamic insights from this method enable informed decisions on material selection and garment structuring, enhancing both functionality and comfort. Beyond sportswear, this approach can be effectively applied to other wearable systems such as medical-grade compression garments for conditions like lymphedema or post-surgical recovery, where precise pressure distribution is critical. It can also guide the ergonomic integration of soft robotic interfaces, such as exosuits, to ensure user comfort and effective biomechanical interaction. Moreover, integrating this anthropometric method into digital garment design workflows can significantly enhance virtual prototyping capabilities. Accurate deformation models allow designers and manufacturers to refine garment prototypes digitally, reducing iteration times, costs, and material waste. The method’s broad applicability thus makes it a valuable tool across sports science, healthcare, rehabilitation, and robotics, offering a foundation for innovative functional textile designs tailored to diverse user needs.

Future work should focus on addressing current methodological limitations and further utilizing this anthropometric method to evaluate the accuracy of 3D biomechanical models. For example, tissue stiffness could be examined through indentation testing, and 3D FE biomechanical models based on magnetic resonance images could be constructed to evaluate tissue deformation with explicit consideration of bone structures. Additionally, incorporating the viscoelastic properties of legging materials will allow for the estimation of long-term dynamic tissue deformation, providing insight into the effects of prolonged garment wear.

While the current use of a 60 Hz vibration protocol provides a controlled and repeatable stimulus to replicate dynamic soft-tissue deformation, this simplified condition does not fully capture the complexity of real-world activities such as running or jumping, which involve variable-frequency loading. Future studies should therefore incorporate a broader spectrum of vibration frequencies or use activity-specific motion capture data to achieve more realistic simulations enabling more accurate predictions of garment-body interactions under various dynamic scenarios.

To improve the generalizability of the simulation models, future research should also include subjects with a broader range of BMIs and body morphologies. Individuals with varying body shapes may exhibit distinct garment-body interaction patterns and tissue deformation behaviors, which are essential for developing compression wear that is both effective and inclusive.

Ultimately, combining the anthropometric method with advanced 3D biomechanical models will facilitate innovative sportswear designs and lead to the development of leggings that fit well and perform optimally during various activities.

## 5 Conclusion

A novel anthropometric method has been developed in this study to determine tissue deformation with the use of sports leggings via body scanning/imaging technology and an analytical model. A Boussinesq solution based on the elastic theory and stress function method is used to predict tissue deformation. The predicted deformation is subsequently validated by using body scanning/imaging technology. The results indicate that the measured and predicted tissue deformations are in agreement with deviations within 1.15 mm for the static condition and 2.36 mm for dynamic conditions, thus validating the accuracy of the novel anthropometric method. The proposed method has significant value for designing leggings by accurately measuring soft tissue deformation during dynamic movement. This method enables designers to make logical and informed decisions, streamline design processes, and customize the end product. These then lead to the creation of leggings that have a better fit, increased wear comfort, and enhanced athletic performance, thus meeting the dynamic needs of users and advancing the field of sportswear design.

## Data Availability

The datasets presented in this article are not readily available because The raw/processed data required to reproduce these findings cannot be shared at this time as the data also forms part of an ongoing study. Requests to access the datasets should be directed to joanne.yip@polyu.edu.hk.
